# Delirium Tremens

**Published:** 1868-01-15

**Authors:** P. S. Macdonald

**Affiliations:** Chicago, Ill.


### DELIRIUM TREMENS.

BY P. S. MACDONALD, M. D., CHICAGO, ILL.

In requesting a place in your valuable journal for the insertion of the following case of *delirium tremens*, I do so, not that I can add any thing new to the category of remedial agents recommended in this disease, or enlarge upon the therapeutic value of those I employed; but, being one of no little interest to myself, and from the fact that it was unusually obstinate to the influence of medical treatment, I thought that a brief description of the case might not prove uninteresting to some of your readers, more especially those who had lately entered as co-laborers into the great field of medical practice.

The patient, James Y.; *æt.* 48; by employment a laborer, and a Canadian by birth, had been for some years addicted to drink, and had during that time been on two previous occasions attacked with *delirium tremens.* On the afternoon of September 11th, I was first requested to see him. His wife informed me that a well-known homœopathist of this city had been in attendance on the patient for some time previous to my being called in, and that she had faithfully administered to her unfortunate husband his *pellets* and *dilutions* without any visible effect or benefit.

On examining the patient I found the leading, well-marked symptoms of *delirium tremens*; pulse soft and compressible, beating 95; tongue moist and covered with a thick, creamy fur; pupils normal; gastritis; urine scant and highly colored; much tremor of the hands, and when the tongue was protruded its tremulous condition also gave evidence of the excited state of the nervous system; his bowels operating every few minutes. Expressing my surprise at the rate in which he was discharging the contents of his bowels, his wife at once threw some light on the matter, by acknowledging that she thought if she could but get the bad whisky cleaned out of him he might get well, and, basing her treatment on the diagnosis she had made, gave him, the day previous, nearly the contents of a box of purgative pills; but the "scouring" did the patient no good—on the contrary, he was worse. I learned at the same time that sleep had not visited his eyes for three nights and four days. In this state of affairs I ordered cold applications to the head, sinapisms to the epigastrium, fomentation of poppy leaves to the abdomen, an enema of beef tea, with *Oleum terebinthinæ* gtt. x., and *Tr. Opii* gtt. xxx., to be given every hour, requesting the *Tr. Opii* to be discontinued in twelve hours, if no tendency to sleep presented itself.

*September* 12*th*, 8 A. M.—Found him more restless; no inclination to sleep; pupils contracted; stomach still intolerant to all kinds of liquids and food; his bowels had evacuated but twice since my previous visit; tongue moist and coated with a whitish fur; pulse about 100, weak and compressible; skin clammy; gave the following prescription:

℞.—Sub. Nit. Bismuth, - - - grs. xxv.

Fiat chart, No. X. One to be given every hour, in small quantities of milk punch and beef tea, ordering at the same time a continuation of the enema, but omitting the *Opii*, deeming it unsafe to push the narcotic further, but in its place added *Tr. Assafœtida*, ℥i. Gave an unfavorable prognosis.

*September* 12*th*, 7 P. M.—No tendency to sleep; pupils much contracted and eyes injected; his mind constantly employed upon those delusions so common in these cases; tremor of hands and tongue increased; pulse rapid and weak; no dejections from the bowels for the last thirteen hours; the urine voided small in quantity and deeply colored; skin clammy, with a copious perspiration; stomach tolerant of small quantities of beef tea and milk punch. Prescribed as follows:

℞.—Bromide Potass., - - - - - ʒi.

Aqua Distil., - - - - - ℥iij.

Mix. S.—Give a teaspoonful every half hour, requesting a continuation of the enemas of beef tea.

*September* 13*th*, 9 A. M.—Still no sleep, and no indications of any; pulse 115, weak and thready; pupils greatly contracted; hands and lower extremities cold and clammy; head and thorax warm; tongue moist and furred; had two evacuations from the bowels since my last visit, and voided his urine twice in the same space of time. I must confess that I was disappointed in not finding the *Bromid. potass.* to have any effect in allaying the excited condition of the nervous system. But the failure ought properly to be attributable to the small quantity of the remedy taken, only four doses, as it could not be continued on account of the irritable condition of the stomach. At this time the general appearance of the patient indicated an approach to collapse, requested warmth to be applied to the lower extremities; an enema of beef tea every hour, to which was added a tablespoonful of brandy; also twenty drops of chloroform every two hours, in some mucillaginous drink, cautioning the nurse to cease giving the latter as soon as any inclination to sleep presented itself.

*September* 13*th*, 8 P. M.—On calling at this hour to see the patient, I was grateful to find many of the obnoxious symptoms, if not entirely gone, were much mitigated, and the patient expressed himself as being much better, if he could but sleep; determined that it should be obtained—I resolved to give the anæsthetic more freely, and to remain with the patient and watch the result; I then gave him one teaspoonful of *Choloroform* in *Glycerine.* In a brief period the effect of this dose was perceptible by a lessened frequency and increased force of the heart's action, and in thirty minutes the harassed and prostrated individual sank into a refreshing slumber, from which he did not awaken for five hours.

*September* 14*th*, 8 A. M.—Found the patient this morning much improved, with every indication of convalescence, except that an acute pain in the ball of the left eye, and over the superciliary ridge annoyed him considerably. Ordered cold applications over the seat of pain, which gave temporary relief. Sustaining treatment continued.

*September* 15*th*, 8 A. M.—Had a tolerable night's rest; left eye much injected; complains of a sharp, lancinating pain in the organ, with diminished power of vision in the right eye. Cold application, as before, gave transitory relief. Ordered the liberal use of *Quinia* and *Iron* for several days. Under this treatment he convalesced gradually, and would have been able to follow his usual occupation, had not *Iritis* developed itself in the left eye, for which he is now under treatment. The patient asserts that prior to his last attack he never experienced any defect of vision or pain in either of his eyes, and never contracted *Syphilis* or *Gonorrhœa*.

